# The impact of physical activity and an additional behavioural risk factor on cardiovascular disease, cancer and all-cause mortality: a systematic review

**DOI:** 10.1186/s12889-019-7030-8

**Published:** 2019-07-08

**Authors:** Jason Lacombe, Miranda E. G. Armstrong, F. Lucy Wright, Charlie Foster

**Affiliations:** 1 Cancer Epidemiology Unit, University of Oxford, Oxford, UK; 2 Centre for Exercise, Nutrition and Health Sciences, School of Policy Studies, University of Bristol, Bristol, UK; 3 Unit of Health Care Epidemiology, Big Data Institute, Nuffield Department of Population Health, NIHR Oxford Biomedical Research Centre, University of Oxford, Old Road, Oxford, OX3 7LF UK

## Abstract

**Background:**

Regular physical activity improves overall health, and has the capacity to reduce risk of chronic diseases and death. However, better understanding of the relationship between multiple lifestyle risk behaviours and disease outcomes is pertinent for prioritising public health messaging. The aim of this systematic review is to examine the association between physical inactivity in combination with additional lifestyle risk behaviours (smoking, alcohol, diet, or sedentary behaviour) for cardiovascular disease, cancer, and all-cause mortality.

**Methods:**

We searched Ovid Medline, EMBASE, and the Cochrane Register from 1 January 2010 to 12 December 2017, for longitudinal observational studies of adults (18+ years) in the general population with a publication date of 2010 onwards and no language restriction. Main exposure variables had to include a physical activity measure plus at least one other lifestyle risk factor. In total, 25,639 studies were identified. Titles, abstracts and full-text articles of potentially relevant papers were screened for eligibility. Data was extracted and quality assessment was completed using a modified Newcastle-Ottawa Scale (NOS).

**Results:**

Across the 25 eligible studies, those participants who reported being physically active combined with achieving other health behaviour goals compared to those who were categorised as physically inactive and did not achieve other positive lifestyle goals, were at least half as likely to experience an incident cardiovascular disease (CVD) event, die from CVD, or die from any cause. These findings were consistent across participant age, sex, and study length of follow-up, and even after excluding lower quality studies. We also observed a similar trend among the few studies which were restricted to cancer outcomes. Most studies did not consider epidemiological challenges that may bias findings, such as residual confounding, reverse causality by pre-existing disease, and measurement error from self-report data.

**Conclusions:**

High levels of physical activity in combination with other positive lifestyle choices is associated with better health outcomes. Applying new approaches to studying the complex relationships between multiple behavioural risk factors, including physical activity, should be a priority.

**Electronic supplementary material:**

The online version of this article (10.1186/s12889-019-7030-8) contains supplementary material, which is available to authorized users.

## Background

An individual’s lifestyle behaviours greatly influence their likelihood of developing and dying of many diseases. In middle to high income countries, physical inactivity, unhealthy eating, smoking, and alcohol consumption, are estimated to contribute to 29% of disease and disability-adjusted life-years lost [1]. According to the World Health Organisation Global Health Risk report, high blood pressure, tobacco use, high blood glucose, physical inactivity, overweight and obesity, high cholesterol, unsafe sex, alcohol use, and low fruit and vegetable intake are the leading risk factors for death in high income countries. Although there are other lifestyle risk factors that have been associated with noncommunicable disease development and death, this review focuses on the most common risk factors which have the biggest effect on disease development and death [2]. These multiple behaviours are also associated with many disease co-morbidities, such as, being overweight or obese, and having high levels of cholesterol, which result in an additional 15% elevated risk, and taken together represent a significant public health problem [1].

The independent effects of each of these behaviours on health outcomes are well-established [3–5]. Therefore, it may seem reasonable to presume that if an individual engages in more than one of these unhealthy behaviours, the potential synergistic interaction amongst them should result in poorer health outcomes. However, the research is limited in assessing the co-distribution of these risk factors, and how the patterns of these risk factors have been evolving over time. Historically, the assessment of multiple behavioural risk factors on health outcomes has primarily used co-occurrence methodologies, which evaluate concurrent but independent engagement in two or more behaviours [6]. In the United Kingdom (UK), 70% of the adult population is estimated to engage in 2 or more of these unhealthy behaviours [1].

Past research has shown that behavioural risk factors are often tackled in policy and clinical contexts as separate issues, to be explored and managed by unique teams and resources [1]. Despite national guidelines and recommendations from the Department of Health [7], the National Institute for Health and Care Excellence [8] and the Royal College of General Practitioners, choosing physical activity as a clinical priority, and its promotion remains sporadic and undervalued compared to other risk behaviours [9]. Indeed, the impact of screening programmes for the primary prevention of cardiovascular disease, including modification of behavioural risk factors, appears limited [10]. However, until we can understand the independent and also synergistic impacts of changing other risk behaviours with physical inactivity the choice of which one to address will remain uninformed. If this were determined, it would help to inform clinicians, researchers, and policy decision makers to better explore multiple lifestyle risks with/for their patients, by understanding their impact on multiple co-morbidities and poor health outcomes.

Here, we aim to systematically evaluate how the association between physical activity in combination with additional lifestyle risk behaviours impacts cardiovascular disease, cancer, and all-cause mortality, and the methods that have been used in previous research to examine the co-distribution of multiple behaviours in adults. Our review is the first global systematic review of adult cohort studies that has examined the associations between physical activity in combination with smoking, alcohol, diet and sedentary behaviour on all-cause mortality, and incident cases or mortality from cardiovascular disease or cancer.

## Methods

### Search strategy and eligibility criteria

We searched Ovid MEDLINE (from 1946), EMBASE (from 1988) and the Cochrane Central Register between 1 January 2010 and 12 December 2017, with no language restrictions, for studies in humans to identify research papers estimating the relationship between physical activity as an exposure variable in combination with one or more other health risk behaviours (smoking, alcohol, diet, or sedentary behaviour), on all-cause mortality, and incident cases or mortality from cardiovascular disease or cancer [7] (Additional file 3). Across the studies, participants were classified as active if those participants met the weekly physical activity guidelines in the country that the study took place. Any participant not meeting the physical activity guidelines were classified as inactive or categorised into a less healthy category. We only focused on the extreme groups (most active group + healthiest profile compared to least active + least healthy profile) when co-occurrence methodologies were used. Our search consisted of terms related to physical activity, smoking, diet, sedentary behaviour, and alcohol consumption, and combined these terms with cardiovascular disease, cancers, mortality and related terms. We also searched reference lists of relevant articles, and of previous reviews. For a detailed list of all search terms used in each database, refer to Additional file 1. The protocol for this systematic review has been published to the PROSPERO register (Reference ID: CRD42017050378 [11]) and the abstract has been presented at the International Society of Behavioral Nutrition and Physical Activity conference.

We included cohort studies of adults (18+ years) from the general population with a publication date of 2010 onwards if they investigated associations between physical activity and at least one additional risk factor (smoking, alcohol, diet, or sedentary behaviour). We only included studies that reported the exclusion of participants with prevalent disease at baseline. Additionally, a start date of 2010 was chosen to align with the date that the World Health Organisation released the most recent global physical activity guidelines [12], and studies with a minimum of 8 years of follow-up were included. We excluded cohorts which were not free-living populations (i.e., hospital patient studies), fitness studies, nutritional supplement, and studies which used a certain type of beverage as a measure of nutrition (i.e., green tea, protein shake). We excluded populations in which studies were restricted to focus on an occupational group, and thus would not be representative of the larger free-living population (i.e., nurse’s, factory workers, students enrolled in a psychology course). We also excluded letters, comments, reviews, meta-analysis, randomised control trials, ecological studies, and animal studies.

### Data extraction and quality assessment

Data extraction was conducted with a standard data collection form describing the characteristics of each study and the main results (Additional file 1). We recorded the following characteristics in the identified studies: author names, name of the paper, cohort name, country, publication year, age at entry, sex, sample size of cohort, confirmation of eligibility criteria (y/n), method used to group lifestyle behaviours, outcome(s), duration of follow-up, method of assessment of physical activity, ascertainment of outcomes, adjustment variables in the multivariate model, regression dilution correction applied (y/n), how was handling of missing data completed, and overall main finding. We also extracted information on whether the study had considered bias, residual confounding, measurement error, and regression dilution. We used hazard ratios or relative risks as a measure of association. The primary exposure variable was physical activity and, as a minimum, one other lifestyle risk factor. Outcomes of interest in this review were all-cause mortality, cardiovascular incidence/mortality, and cancer incidence/mortality.

We assessed study quality using a modified Newcastle-Ottawa Scale (NOS) for cohort studies [13]. The NOS assesses the quality of nonrandomised studies. A point system has been developed in which a study is judged on, the selection of the study groups, the comparability of the groups, and the ascertainment of either the exposure or outcome of interest for cohort studies. The NOS is recommended by the Cochrane Collaboration as an assessment tool for cohort studies to assess risk of bias. The content validity of the NOS has been established based on a critical examination by several experts. Using NOS for cohort studies, the maximum score a study can receive is 9 points. For this review, we then added additional criteria, to assess the strength of the physical activity measure that was used in more detail, and to explore the control variables that studies used. Using the modified scale, a study could be awarded a maximum of 10 points. Relating to the question on ascertainment of exposure, the question was modified, and studies were given a point if they used either structured interview, or a validated self-report measure. Studies were awarded an additional point (bringing the total up to 10) if they controlled for age, sex, or a measure of socio-economic status/education.

### Data synthesis

Overall, studies included in this review were too heterogeneous in terms of participant characteristics, intervention design, and outcome measures to allow for a detailed meta-analysis, thus data was synthesised descriptively. Some additional analysis to examine the highest-quality studies in more detail was conducted using the statistical program R (version 3.3.3) and a forest plot was created.

To be included in the preliminary analysis (Table 1), all studies had to have at minimum 8 years of follow-up and a measure of physical activity with an additional exposure variable. Studies also needed to score 8 points or higher on the modified NOS. To take this further, we did more detailed analysis on studies which met these criteria, and additionally used a validated self-report physical activity measure or objective measure. We present all the results in one direction examining the healthiest profile against the least healthy profile. As a result, when necessary the inverse risk ratio has been calculated. In general for all results, the risk ratios are presented and a forest plot was created to visualise the risk relationship between lifestyle behaviour, and disease/death outcomes with the healthiest lifestyle group as the reference compared to the least healthy lifestyle group. We also plotted the 95% Confidence Intervals for each study of interest. For studies which presented risk associations and confidence intervals where the least healthy group was used as the reference, we computed and plotted the inverse risk and confidence intervals.

**Table 1:** Summary of high quality studies (*n* = 25)

Author, year	Study name	Country	Study length (years)	Number in cohort	Age at baseline (mean or range) years	Validated physical activity?	Physical activity measurement	Adjustments	Exposure variables	Outcome
Shaw et al., 2014 [18]	Level of Living Survey (LNU) and the Swedish Panel Study of Living Conditions of the Oldest Old (SWEOLD)	Sweden	13	1682	44	Structured interview	Index summarising activity over the past 12 months. Respondents engaging in gardening, hunting, dancing, and any type of sport “sometimes” received one point and two points if reported as “often”. Two points were also given for any person who engaged in a sport regardless of frequency. If one had a score of less than two they were defined as inactive.	Age, gender, education, and occupational status. Also, to account for the potential influence of poor health on smoking and physical inactivity behavior, all models controlled for health status in 1981, including measures of circulatory problems mobility problems, and psychological problems.	PA and smoking	All-cause mortality
Prinelli et al., 2015 [14]	Intervention title not mentioned.	Italy	17.4	974	40–74	Not validated	PA was assessed by questionnaire asking about the frequency that one played sports and a binary score was created. Physically active if they were engaged in at least one sport, and physically inactive if they did not play sports.	Age, sex, education level, BMI, time spent watching television, and energy expenditure.	PA, diet, and smoking.	All-cause mortality
Behrens et al., 2013 [29]	NIH-AARP Diet and Health Study.	USA	12.5	170,672	62.5	Not validated	Looked at vigorous and moderate PA. Used a cut-off of 3 times of vigorous PA per week to define adherence to the vigorous PA recommendations. Used a cut-off of 3 h/week of moderate activity to approximate adherence to the moderate PA recommendations. People were assumed to meet the American College of Sports Medicine guidelines if they met 1 of the moderate or vigorous criteria.	Age, marital status, education, race/ethnicity, body height, hip circumference, and alcohol intake.	PA, abdominal leanness, smoking, and diet. Sub-analysis included alcohol consumption	All-cause mortality
Shaw & Agahi, 2012 [30]	Health and Retirement Study (HRS)	USA	10	19,662	Not mentioned because combined multiple cohorts.	Not validated	Were asked, “On average over the last 12 months have you participated in vigorous PA or exercise three times a week or more?” If they answered yes, they were classified as active, and no, inactive.	Age, gender, race, education, household income, and marital status. Also, to control for potential health conditions, they controlled for self-rated health, self-reported presence of 5 serious chronic conditions, a count of functional limitations, and weight status.	PA, smoking, alcohol consumption	All-cause mortality
Carlsson et al., 2013 [31]	Intervention title not mentioned.	Sweden	11	4232	60	Not validated	Leisure time PA during the past year was categorised as inactive, light PA if at least 2 h/week, moderate PA if at least once or twice per week, and intense PA if 3+ times per week. Healthy PA score was at least once per week.	Age (everyone was 60 years old), sex, education level and BMI.	PA, smoking, and diet.	All-cause mortality and CVD
Hulsegge et al., 2016 [39]	The Doetinchem Cohort Study	Netherlands	9.8	5263	46	Validated	Hours/week of spent on PA was summed. Only PA’s with a MET value of 4.0 or higher were included, which is in line with the Dutch PA recommendations.	Age, sex, highest educational level achieved during follow-up, and employment status in model	PA, diet, smoking, and alcohol consumption	All-cause mortality and CVD
Petersen et al., 2015 [32]	Diet Cancer and Health Study	Denmark	14	6768	50–64	Not validated	Examined the average number of hours/week spent in the past year on PA in leisure, moderate, and high intensity time during summer and winter months, which were averaged. Being physically active for at least 30 min/day at moderate intensity was set as the cut-off following the World Cancer Research Fund and Nordic recommendations.	Age, civil status, and length of education.	PA, smoking, alcohol consumption, waist circumference and diet.	All-cause mortality, CVD, and Cancer
Yun et al., 2012 [20]	Intervention title not mentioned.	Korea	10.3	59,941	30–84	Structured interview	Participants activity levels were assessed via interview, and participants were given a score of 1 (binary) if they reported PA (never, < 3 times per/week).	Stratified by sex and adjusted for age, income, and education	PA, smoking, alcohol consumption, BMI. Also, subgroup analysis included fruit and vegetable consumption	All-cause mortality and Cancer
Kvaavik et al., 2010 [19]	Health and Lifestyle Survey (HALS)	United Kingdom	20	4886	43.7	Structured interview	PA was assessed by number of leisure time exercise activities that one had performed during the past fortnight and time spent doing these activities. Poor PA was defined as spending little or no time on PA (< 120 min during 1 week).	Age, sex, occupational social class, BMI, blood pressure, and the other three health behaviours.	PA, smoking, diet, and alcohol.	All-cause mortality, CVD, and Cancer
McCullough et al., 2011 [36]	The CPS-II Nutrition Cohort	USA	14	111,966	Men 63.6 years & women 61.9 years.	Not validated	Participants reported average time per week spent in 7 recreational activities. These were then converted into MET scores. MET-hours/week less than 8.75 received a score of 0, 8.75 to < 17.5 earned a score of 1, and ≥ 17.5 earned a score of two for reaching preferable levels according to the American Cancer Society.	Age, education, smoking history.	PA, BMI, diet, and alcohol consumption	All-cause mortality, CVD, and Cancer
Larsson et al., 2016 [25]	Cohort of Swedish Men (COSM) and the Swedish Mammography Cohort (SMC)	Sweden	13	64,679	59.3	Validated	PA was assessed in time spent on various activities during the previous year. Researchers added up time per week spent engaged in walking/bicycling and exercise. Those participants with ≥150 min/wk. of PA were classified as active.	Age, education, family history of myocardial infarction before 60 years of age, aspirin, and history and diagnosis of hypertension, hypercholesterolemia, diabetes mellitus, and atrial fibrillation before baseline.	PA, smoking, BMI, and diet	Heart failure
Kabat et al., 2015 [15]	NIH-AARP study	USA	10.5	476,396	50–71	Not validated	PA assessment was based on PA was based on 4 levels of strenuous activity (never/rarely/1–3 times per month, 1–2 times per week, 3–4 times per week, and ≥ 5 times per week).	Age, education, race, 14-level smoking variable, marital status. In addition, they examined the association of adherence with risk of cancer at individual sites in men and women separately and, for sites with smaller numbers, in both sexes combined.	PA, BMI, diet, and alcohol consumption.	All-cause mortality and Cancer
Larsson et al., 2014 [33]	Swedish Mammography Cohort	Sweden	10.4	31,696	49–83	Not validated	Women reported on average how many minutes per day they had walked/bicycled, and how many hours per week they had exercised. Binary PA was created and a low risk PA behaviour was considered to include both low to moderate activities (walk/cycling ≥40 min/day), or more vigorous PA (exercise ≥1 h/week).	Age, educational, use of aspirin, diabetes, atrial fibrillation, family history of myocardial infarction before the age of 60 years, total energy intake, and non-recommended foodscore.	PA, diet, alcohol consumption, smoking, and BMI	Stroke
Akesson et al., 2014 [26]	Intervention title not mentioned.	Sweden	11	20,721	45–70	Validated	Low risk PA included men who walked or cycled for ≥40 min/day and exercised ≥1 h/week.	Age, level of education, marital status, family history of MI, use of aspirin, non-Recommended Food Score, and energy intake.	PA, smoking, diet, alcohol consumption and waist circumference.	MI
Chomistek et al., 2013	Women’s Health Initiative Observational Study	USA	12.2	71,018	50–79	Not validated	MET hours/week was computed as a sum of frequency, duration, and intensity of PA. Women were classified into 4 categories: inactive (≤1.7 MET hours/week), low PA (1.8–8.3 MET hours/week), medium PA (8.4–20 MET hours/week), and high PA (> 20 MET hours/week).	Stratified by age, and covariates included race/ethnic group, family income, education, marital status, smoking, parental history of premature MI, depression, alcohol intake, hours of sleep, and intake of total calories, saturated fat, and fiber.	PA and sitting time	CVD
Wang et al., 2011 [35]	Five independent cross-sectional, population-based health examination surveys (FINRISK)	Finland	14.1	38,075	25–64	Not validated	PA was merged into 3 categories: ‘low’, corresponding to light levels of both occupational and leisure time PA; ‘moderate’, corresponding to moderate or high level occupational or leisure time PA; and, ‘high’, corresponding to moderate or high level of both occupational and leisure time PA. This was then dichotomized into PA (low versus moderate or high).	Age, study year, education, systolic blood pressure, total cholesterol, and history of myocardial infarction, valvular heart disease, diabetes, and using antihypertensive drug	PA, smoking, BMI, and vegetable consumption	Heart failure
Kirkegaard et al., 2010 [27]	Diet, Cancer and Health Cohort Study	Denmark	9.9	55,487	50–64	Validated	Participants reported their PA via reporting avg. number of hours/week spent the past year engaged in leisure activity, or occupational activity. Participants were deemed active if they reported > 30 mins/day of activity or had a job with light manual activity or heavy manual activity.	Age, education, use of non-steroidal anti-inflammatory drugs, hormone replacement therapy among women, and history of cancer in first degree relatives.	PA, waist circumference, smoking, alcohol consumption, and diet.	Colorectal cancer
Nomura et al., 2016 [28]	The Black Women’s Health Study	USA	13.9	49,103	21–69	Validated	PA was scored against the World Cancer Research Fund/American Institute for Cancer Research recommendations.	Age, geographic region, caloric intake, smoking, family history of colorectal cancer, education, menopausal status, diabetes, insulin usage, aspirin usage, colonoscopy, and sigmoidoscopy.	PA, smoking, and diet	Colorectal cancer
McKenzie et al., 2015 [37]	European Prospective into Cancer and Nutrition (EPIC) cohort study	Europe (mix of countries)	10.9	242,918	53.2	Not validated	Healthiest behaviour was defined as scoring in the top quintile of PA based on recreational and household MET tasks.	Height, age at menarche, age at full term pregnancy, education, oral contraceptive use, hormone replacement therapy use, breastfeeding, total energy intake excluding alcohol	PA, smoking, alcohol consumption, BMI, and diet.	Breast Cancer
Eriksen et al., 2015 [17]	UK SABRE Study	United Kingdom	21	2096	40–69	Not validated	PA was computed as the total weekly energy expended in sporting activities, cycling, walking, and in other strenuous activity during leisure time. Being active and meeting the cutoff was defined as those undertaking moderate exercise for 5+ hours per week, or vigorous exercise for ≥2.5 h/week.	Age, sex, systolic and diastolic blood pressure, hypertension treatment, total cholesterol, HDL-cholesterol, BMI, social class, employment status, and occupational physical activity.	PA, smoking, alcohol consumption, and fruit and vegetable consumption	CVD
Vergnaud et al., 2013 [38]	European Prospective into Cancer and Nutrition (EPIC) cohort study	Europe (mix of countries)	12.8	121,443	25–70	Not validated	PA was scored against the World Cancer Research Fund/American Institute for Cancer Research recommendations. We looked at men only.	Stratified for sex, age, and center and adjusted for educational, smoking, and intensity of smoking. Additional adjustment for total energy intake.	PA, BMI, diet, and alcohol consumption	All-cause mortality, CVD, and Cancer
Fazel-taber Malekshah et al., 2016 [16]	Golestan Cohort Study (GCS)	Iran	8.0	40,708	40–75	Not validated	For PA, a low risk group was those that reported at least 30 min/day of moderate and vigorous PA on average.	Adjusted for age, gender, resident area, ethnicity, socioeconomic status (Wealth Score), marital status, education, alcohol consumption, and opium use.	PA, smoking, and alternative eating index.	All-cause mortality, CVD, and cancer.
Zhang et al., 2017 [21]	Shanghai Men’s Health Study	China	9.3	59,503	40–74	Validated	People were categorised as healthy if they engaged in > = 150 min/week of moderate-to-vigorous PA.	Adjusted for age, occupation, education, income, and history of diabetes mellitus, and colorectal cancer family history of first degree relatives.	PA, waist hip ratio, smoking, alcohol consumption, and dietary behaviour.	Colorectal cancer
O’Donovan et al., 2017 [22]	Health Survey for England (HSE) and Scottish Health Survey (SHS)	UK	9.4	106,341	47	Validated	Asked questions about PA frequency and duration in activity (domestic PA, light-intensity, moderate-intensity) and type/intensity for sports/exercise. MET-hours were calculated and 3 categories of PA were made (≥60 min/wk., < 60 min/wk., and none).	Adjusted for age, sex, occupation, and longstanding illness.	PA and smoking	All-cause mortality, CVD, and cancer.
Larsson et al., 2017 [23]	Cohort of Swedish Men and Swedish Mammography Cohort	Sweden	15.3 for men and 15.7 for women	33,454 men and 30,639 women	45–79	Validated	PA and sedentary behaviour was calculated by asking about previous year. Participants who had recorded on average at least 150 min per week of activity were categorised as healthy and those with < 150 min per week of activity as unhealthy.	Adjusted for age, education, family history of MI and cancer, asprin use, sedentary time, BMI, and history of diabetes, hypertension and hypercholesterolemia.	PA, smoking, alcohol consumption, and a healthy diet	All-cause mortality

*BMI* Body mass index, *CVD* Cardiovascular disease, *MI* Myocardial infarction, *PA* physical activity, *UK* United Kingdom, *USA* United States of America

## Results

### Literature search

**Fig. 1:**
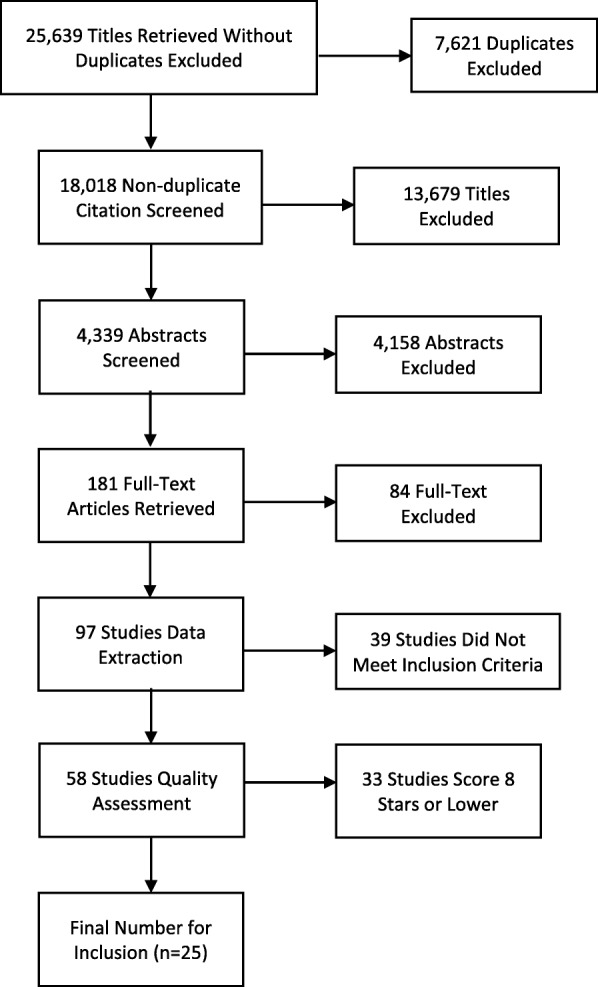
A Preferred Reporting Items for Systematic Reviews and Meta-Analyses (PRISMA) checklist is presented showing study inclusion decisions

From an initial 25,639 studies retrieved, and following removal of duplicates (*n* = 7621), we screened 18,018 titles. Exclusion by title for all returned results (JL), and a 10% double review of titles was completed by 2 independent assessors (JL and MA). This resulted in 4339 abstracts to be reviewed (JL) and all abstracts were completed (JL and CF) by two independent assessors. This left 181 full-text articles, which were read in-detail (JL), and 84 of these were then excluded because they did not meet inclusion criteria. Data extraction including the characteristics of the samples, methods, and main results was completed for the remaining 97 eligible studies (JL). From data extraction, 39 studies did not meet our criteria, and were excluded. A total of 58 studies were included for the final step, which comprised quality assessment completed independently by 2 assessors (JL & FLW). For the final analysis, we included only studies which scored at least 8 points or higher out of 10 (*n =* 25). A Preferred Reporting Items for Systematic Reviews and Meta-Analyses (PRISMA) flow diagram is presented showing study inclusion decisions (Fig. 1) and a PRISMA checklist is presented in the Additional file 2.

### Included studies and measures

Table 1 summarizes the characteristics of the 25 studies included. Studies were conducted in the USA (*n* = 6), Sweden (*n* = 6), multiple European countries (*n* = 2), Denmark (*n* = 2), United Kingdom (*n* = 3), Italy (*n* = 1), Netherlands (*n* = 1), Korea (*n* = 1), China (*n* = 1), Iran (*n* = 1), and Finland (*n* = 1). The number of participants in each study ranged from 974 [14] to 476,396 [14]. The minimum study length of follow-up was 8.1 years [15], and the maximum was 21 years [16]. In total, 3 studies used a structured interview to assess physical activity behaviour [17–19], 8 used a validated self-report questionnaire [19–26], and 14 did not use a validated questionnaire [14–16, 27–37]. No study included an objective measure of physical activity (i.e., accelerometry or pedometers).

## Outcomes

### All-cause mortality

There was strong evidence that more physical activity and choosing better lifestyle behaviours led to longer survival in all 15 high quality studies examining all-cause mortality. However, the level of risk varied across different combinations of behavioural patterns. All studies found that a high adherence to positive factors including higher levels of physical activity, not smoking, eating healthy, and limited sedentary behaviour and alcohol consumption were strongly associated with reduced risk of all-cause mortality after long-term follow-up, and that this association was even stronger when these positive behaviours were combined [14, 17–19, 21–23, 27–31, 35, 37].

For example, Kvaaik and Colleagues (2010) found that after 20-years of follow-up, those in the healthiest category for all 4 lifestyle behaviours versus those in the least healthy category of lifestyle behaviours had a total mortality risk of 0.29 (CI = 0.19–0.43) [18]. Similarly, Prinelli and Colleagues (2015) followed subjects for an average of 17.4 years, and found that those with 1, 2, or 3 healthy behaviours had a significantly reduced risk of death of, 39, 56, and 73% respectively [14]. Petersen and Colleagues (2015) and McCullough and Colleagues (2011) both followed men and women for 14 years on average and found that every additional health recommendation adhered to, had a greater protective effect against mortality [31, 35]. Comparing lifestyle scores, Fazel-taber Malekshah and colleagues (2016) reported on observations from 40,708 participants, adults in the most healthy group had a significantly reduced risk of morality (RR = 0.68; CI = 0.54–0.86) compared to those in the least healthy group [15].

When examining the associations by different subgroups, McCullough and Colleagues (2011) limited their study to only non-smokers to explore in more detail the impact that following other prevention guidelines besides tobacco avoidance has on a cohort [35]. When examining all-cause mortality outcomes by sex, Larsson and Colleagues (2017) found that those with all four positive health behaviours compared to 0 had a reduced risk for men (RR = 0.47; CI = 0.44–0.51) and women (RR = 0.39; CI = 0.35–0.44) [22]. Similarly, Yun and colleagues (2012) found men and women with 4 healthy lifestyle factor points including high levels of physical activity compared to no healthy lifestyle factor points and no physical activity reported had a 0.50 (0.23–1.08) [19]. Behrens and Colleagues (2013) included a measure of adiposity in their lifestyle score and found that these results held, even with an additional measure [28]. Physical activity significantly reduced risk of mortality independently (RR = 0.86; CI = 0.84–0.89) with the larger the number of positive behaviours adhered to, the lower the risk, estimating that 33% of deaths were prevented if subjects adhered to all positive health behaviours. Examining the dual-associations between physical activity duration and history of smoking, O’Donovan and colleagues (2017) found those who exercised for over 60 min/week and were never smokers had a significantly reduced mortality risk compared to those who did no exercise and were current smokers, (RR = 0.29; CI = 0.24–0.36) [21].

### Cardiovascular disease

A total of 14 high quality studies included a CVD outcome. This included 10 which examined CVD broadly [15, 16, 18, 21, 30, 31, 33, 35, 37, 38], 1 stroke incidence [32], 1 myocardial infarction [25], and 2 heart failure [24, 34]. Similar to the all-cause mortality findings, there was evidence that engaging in regular physical activity, and additional healthy behaviours was associated with a reduced risk for developing and dying of cardiovascular outcomes, and these finding were consistent across gender, age, and populations sampled. After 21 years of follow-up in a sample of 2096 participants, Eriksen and Colleagues (2015) found that the population attributable fraction for Coronary Heart Disease (CHD) and CVD amongst both Europeans (*n* = 1090) and South Asians (*n* = 1006) was 43% for CHD and 28% for CVD in Europeans and 63% for CHD and 51% for CVD in South Asians who did not have any of the four healthy behaviours surveyed at baseline [16]. Kvaanik and others (2010) looked at the influence of lifestyle on CVD over 20 years and found that the population attributable risk for CVD was 30%, and compared to those who did no physical activity and had the least healthy profile, individuals in the healthiest category had the greatest reduced risk 0.32 (CI = 0.15–0.69) [18]. In 40,708 participants comparing lifestyle scores over an 8-year follow-up period, those in the healthy group had a significantly reduced risk of CVD mortality (RR = 0.53, CI = 0.37–0.77) compared to those in the unhealthy group [15]. Hulsegge and Colleagues (2016) found that independent of baseline lifestyle behaviour reporting when re-measured later, each decrement in lifestyle factors was associated with a 35% higher risk of CVD, and individuals who maintained their healthy lifestyle over time (4 to 5 factors) had 2.5 times lower risk of CVD (HR = 0.43, CI = 0.25–0.63) compared to those who maintained an unhealthy lifestyle profile (0 to 1 factors) [23].

Examining various sub-groups, Petersen and Colleagues (2015) found a significant risk reduction amongst those men and women who achieved all five lifestyle behaviour points compared to those who did not [31]. For CVD mortality, the adjusted hazard ratio for those in the category of 4–5 positive lifestyle behaviours compared to those with 0 was 0.20 (CI = 0.14–0.0.29) for men and 0.21 (CI = 0.11–0.41) for women. In a population-based cohort of 60-year-old men (*n* = 2039) and women (*n* = 2193) after 11 years of follow-up, for those categorised as very healthy compared to unhealthy, the respective hazard ratios were 0.25 (CI = 0.15–0.44) for men and 0.35 (CI = 0.23–0.54) for women [30]. Examining men only (we excluded women because one of the scoring criteria included breastfeeding), Vergnaud and others (2013) found that after 12.8 years, men in the healthiest category compared to the least healthy category (RR = 0.64, CI = 0.50–0.82) were less likely to die of CVD [37]. Moreover, the largest study cohort (*n* = 111,966) found that achieving more recommended behaviours categorized as healthy compared to least healthy had a reduced risk of CVD mortality in both men (RR = 0.52, CI = 0.45–0.59) and women (RR = 0.42, CI = 0.35–0.51) over 14 years of follow-up [35].

When examining the dual-association between sedentary behaviour, specifically sitting time, and physical activity in women, Chomistek and Colleagues (2013) found a non-statistically significant interaction between sitting time and physical activity with CVD (*p*_*interaction*_ = 0.94) [33]. Similarly, O’Donovan and Colleagues (2017) examined the dual-associations between physical activity and smoking behaviour in 106,341 participants and found that after 9.4 years, those who were regular exercisers of over 60 min a week and did not smoke had a reduced risk of CVD mortality (RR = 0.27, CI = 0.18–0.42) compared to those who did not exercise and were current smokers [21].

Similar results were evident for heart failure when examining men and women separately [34]. Wang and Colleagues (2011) examined 18,346 men and 19,729 women and followed them for a median of 14.1 years. They found that for both men and women, having a healthier lifestyle profile resulted in a reduced risk of heart failure [34]. Having all 4 lifestyle factors compared to none, resulted in a hazard ratio of 0.31 (CI = 0.17–0.56) for men and, 0.19 (CI = 0.09–0.40) for women. In a sample of 33,966 men and 30,713 women followed for 13 years, the relative risks for heart disease in the healthiest lifestyle profile compared to the least healthy profile, which included those with 0 healthy lifestyle risk factors, was 0.38 (0.28–0.53) in men and 0.28 (0.19–0.41) in women [24]. For stroke, it was found that compared to the least healthy lifestyle profile (no lifestyle points), those with the healthiest profile (5 lifestyle points) had a relative risk of 0.38 (CI = 0.20–0.73) [32]. Finally, examining incident myocardial infarction in 20,721 men, followed for 11 years, having all 5 positive lifestyle points compared to none gave a relative risk of 0.14 (CI = 0.04–0.43), and these researchers concluded that this combination of positive behaviours could have prevented 79% (CI = 0.34–0.93%) of the events in this study cohort [25].

### Cancers

A total of 12 studies assessed cancer as an outcome. Eight studies focused on cancer broadly [15–17, 21, 27, 31, 35, 37]. Three studies examined colorectal cancer [20, 26, 39], and one study examined breast cancer [36].

In general, engaging in a higher number of healthy behaviours compared to less, led to greater protection against most cancers. Kabat and Colleagues (2015) followed 476,396 participants for 10.5 years on average, during which time, 73,784 people had a first incident cancer diagnoses and 16,193 people died of cancer [27]. Similarly, in 40,708 participants after 8-years of follow-up, those in the healthy group compared to the least healthy group had a reduced risk of cancer mortality (RR = 0.82, CI = 0.53–0.86) [15]. Moreover, Kaavik and colleagues followed UK adults for 20 years where 18 deaths of 4886 deaths were attributed to cancer and they found that those participants with all positive health behaviours and those taking more physical activity versus those with no positive health behaviours and reporting no physical activity had a hazard ratio of 0.29 (CI = 0.15–0.60) [18].

Looking at subgroups, McCullough and others (2011) examined 111,966 non-smoking men and women and the relative risk for those in the healthiest category of lifestyle behaviour (7–8) versus those in the least healthy category (0–2) and found that risk was 0.70 (CI = 0.61–0.80) for men and 0.76 (CI = 0.65–0.89) for women [35]. This was similar to Vergnaud and Colleagues (2013) where men’s adjusted risk was 0.86 (CI = 0.69–1.07) for cancer after a median of 12.7 years of follow-up [37]. Additionally, after 10.3 years of follow-up of 59,941 Koreans, Yun and colleagues found that the risk for cancer mortality was 0.42 (CI = 0.35–0.69) in men and 0.50 (0.23–1.08) in women [19]. Petersen and colleagues (2015) explored cancer mortality risk in Danish men and women over a 14-year period and found that adherence to 4–5 positive lifestyle behaviours versus 0 positive lifestyle behaviours gave an adjusted hazard ratio of 0.33 (0.26–0.42) for cancer mortality in men and 0.41 (0.29–0.58) in women [31]. Lastly, O’Donovan and Colleagues (2017) examined the dual-associations between physical activity and smoking behaviour and found that regular exercisers who did not smoke had a greater reduced risk of cancer mortality (RR = 0.30, CI = 0.22–0.41) compared to those who never exercised and were current smokers [21].

Three cohort studies examined colorectal cancer specifically. In a sample of 59,503 men followed for 9.28 years, Zhang and Colleagues (2017) found that each increment in score in having a healthier lifestyle was associated with a 17% reduced risk of colorectal cancer (HR = 0.83; CI = 0.78–0.89), 27% for rectal cancer (HR = 0.73; CI = 0.66–0.82), and 10% for colon cancer (HR = 0.90; CI = 0.83–0.99) [20]. Kirkegaard and Colleagues (2010) found that higher physical activity levels and choosing more positive lifestyle behaviours was associated with a lower risk of colorectal cancer in a sample of 55,487 men and women, incidence rate ratio 0.89 (CI = 0.82–0.96) [26]. However, Nomura and Colleagues (2016) when examining incidence in African American women, found that adherence to more positive behaviours were not associated with colorectal cancer risk [39]. Lastly, the one cohort study examining breast cancer risk in postmenopausal women (*n* = 242,912) found that having a higher score of healthier behaviours (score of 4 compared to 1) reduced the risk of breast cancer incidence after a median of 10.9 years of follow-up (adjusted hazard ratio = 0.74, CI = 0.66–0.83) [36].

### High quality studies

When we included more stringent criteria, restricting to studies with 8+ years of follow-up, and the use of a structured interview or a validated physical activity measure, we had 11 remaining studies (Table 2). Outcomes included in the final analysis were 7 for all-cause mortality, 6 for cardiovascular diseases, and 7 for cancers. Additionally, some studies performed subgroup analysis by sex, and others targeted only one sex specifically. Overall, trends suggested that engaging in a greater number of positive health behaviours resulted in reduced risk of death, and being less likely to develop and die of cardiovascular diseases, and cancers.

**Table 2:** Description of studies with a validated physical activity measure and 8+ years of follow-up (*n* = 11)

Author(s), year	Physical activity (PA) in best group	Best Group	Worst Group	Main Finding	Effect of increasing PA on risk
Shaw and Agahi, 2014 [18]	Participants needed 2 PA points. Received 1 point if they reported that they ‘sometimes’ did gardening, hunting, and dancing and 2 points if they reported ‘often’ during the past 12 months. Any sports participation (often or sometimes) was rewarded 2 points.	Active/Non-smoking (reference)	Discontinued activity/ Persistent smoking	All participants with high PA who were non-smokers had a significantly reduced mortality risk compared to no PA and smokers, 0.48 (CI = 0.36–0.67)^a^.	↓ Mortality risk
Hulsegge et al., 2016 [24]	PA ≥3.5 h/week at both waves.	4 to 5 healthy factors at baseline and follow-up	0 to 1 factors at baseline and follow-up	Each increase in number of healthy lifestyle factors at a later date led to a reduced risk of CVD and all-cause mortality. Individuals who maintained a healthy lifestyle profile had a 57% lower risk of CVD, 0.43 (CI = 0.25–0.63)^a^, and 60% lower risk of all-cause mortality, 0.40 (CI = 0.22–0.73)^a^ compared to those with an unhealthy lifestyle.	↓ All-cause mortality and CVD
Yun et al., 2012 [20]	PA ≥3 times/week	0 lifestyle factor points	4 lifestyle factor points	In men and women, compared to those having all 4 of the poor lifestyle factor points, those with 0 poor lifestyle factor points and high PA men had a 0.42 (CI = 0.35–0.69)^a^ reduced risk of cancer mortality and women had a 0.50 (0.23–1.08)^a^ reduced risk. For all-cause mortality, men had a 0.50 (CI = 0.40–0.63)^a^ reduced risk, and women had a 0.48 (CI = 0.28–0.82)^a^ reduced risk.	↓ All-cause mortality and Cancer mortality
Kvaavik et al., 2010 [19]	PA ≥2 h/week	0 lifestyle factor points	4 lifestyle factor points	Compared to those in the least healthy group (4 points and no PA) those with all healthy lifestyle beahviours and high PA (0 points) was associated with a reduced risk of death (all—cause mortality, CVD, and cancer). Morality risk was 0.29 (CI = 0.19–0.43)^a^ for death by all-causes, 0.32 (CI = 0.15–0.69^a^ for CVD and 0.29 (CI = 0.15–0.60)^a^ for cancer in the healthiest group (0 points) group at baseline compared to the least healthy (4 points).	↓ Mortality for all-causes, CVD, and Cancer
Larsson et al., 2016 [25]	PA ≥150 min/week	4 lifestyle factor points	0 lifestyle factor points	Compared with those with no healthy lifestyle factors, the multivariable relative risks of heart failure for those with all 4 lifestyle factors were 0.38 (CI = 0.28–0.53)^a^ in men and 0.28 (CI = 0.19–0.41)^a^ in women	↓ Risk of heart failure
Åkesson et al., 2014 [26]	Walking/bicycling ≥40 min/day and exercising ≥1 h/week	5 lifestyle factor points	0 lifestyle factor points	Compared with those with 0 lifestyle factor points, men with all 5 lifestyle factor points had a relative risk of 0.14 (95%CI: 0.04–0.43)^a^. This profile could prevent 79% (CI = 34–93%) of all myocardial events based on this study population’s characteristics.	↓ Myocardial infarction risk
Kirkegaard et al., 2010 [27]	> 30 mins of moderate activity/day or light or heavy occupational activity each day	5 lifestyle factor points	0 lifestyle factor points	Compared with the worst lifestyle behavior’s, participants who had the best behaviors had a 58% lower risk of developing colorectal cancer, but this was non-significant due to the wide confidence intervals (0.42, CI: 0.13–1.32)^a^.	NS Colorectal cancer incidence
Nomura et al., 2016 [28]	≥3–4 h/week vigorous PA or ≥ 5–6 h/week walking.	7 lifestyle factor points	0 lifestyle factor points	Regardless of the modeling approach used, adherence to a greater number of recommendations was not significantly associated with reduced colorectal cancer risk, 1.01 (95%CI = 0.82–1.24). Similar results were observed for colon cancer, 1.06 (95%CI = 0.84–1.35).	NS Colorectal cancer risk NS Colon cancer risk
Zhang et al., 2017 [21]	≥150 min/week of moderate-to-vigorous-intensity PA	4–5 lifestyle factor points	0 lifestyle factor points	Compared with the least healthy lifestyle group, those in the healthies lifestyle group had a hazard ratio of 0.65 (95%CI = 0.47–0.90)^a^ for colon cancer, 0.35 (95%CI = 0.24–0.52)^a^ for rectal cancer, and 0.50 (95%CI = 0.39–0.65)^a^ for colorectal cancer.	NS colon cancer risk ↓ Rectal cancer risk ↓ Colorectal cancer risk
O’Donovan et al., 2017 [22]	≥60 min/week of PA	≥60 min/week of PA and non-smokers	No exercise and current smokers	Those who exercised for over 60 min/week and were never smokers compared to those who did no exercise and were current smokers had a 0.29 (CI = 0.24–0.36)^a^ reduced risk for all-cause mortality, 0.27 (CI = 0.18–0.42)^a^ for CVD mortality and 0.30 (CI = 0.22–0.41)^a^ for cancer mortality.	↓ All-cause mortality, ↓ CVD mortality, and ↓ cancer mortality
Larsson et al., 2017 [23]	≥150 min/week of PA	4 lifestyle factor points	0 lifestyle factor points	Compared to the least healthy group, those in the healthiest group the hazard ratio for all-cause mortality for men was 0.47 (95%CI = 0.44–0.51)^a^ and for women this was 0.39 (95%CI = 0.35–0.44)^a^.	↓ All-cause mortality

*CVD* cardiovascular disease, *PA* physical activity, *NS* non-significant

^a^computed the inverse risk and confidence intervals for Forest plot

**Fig. 2:**
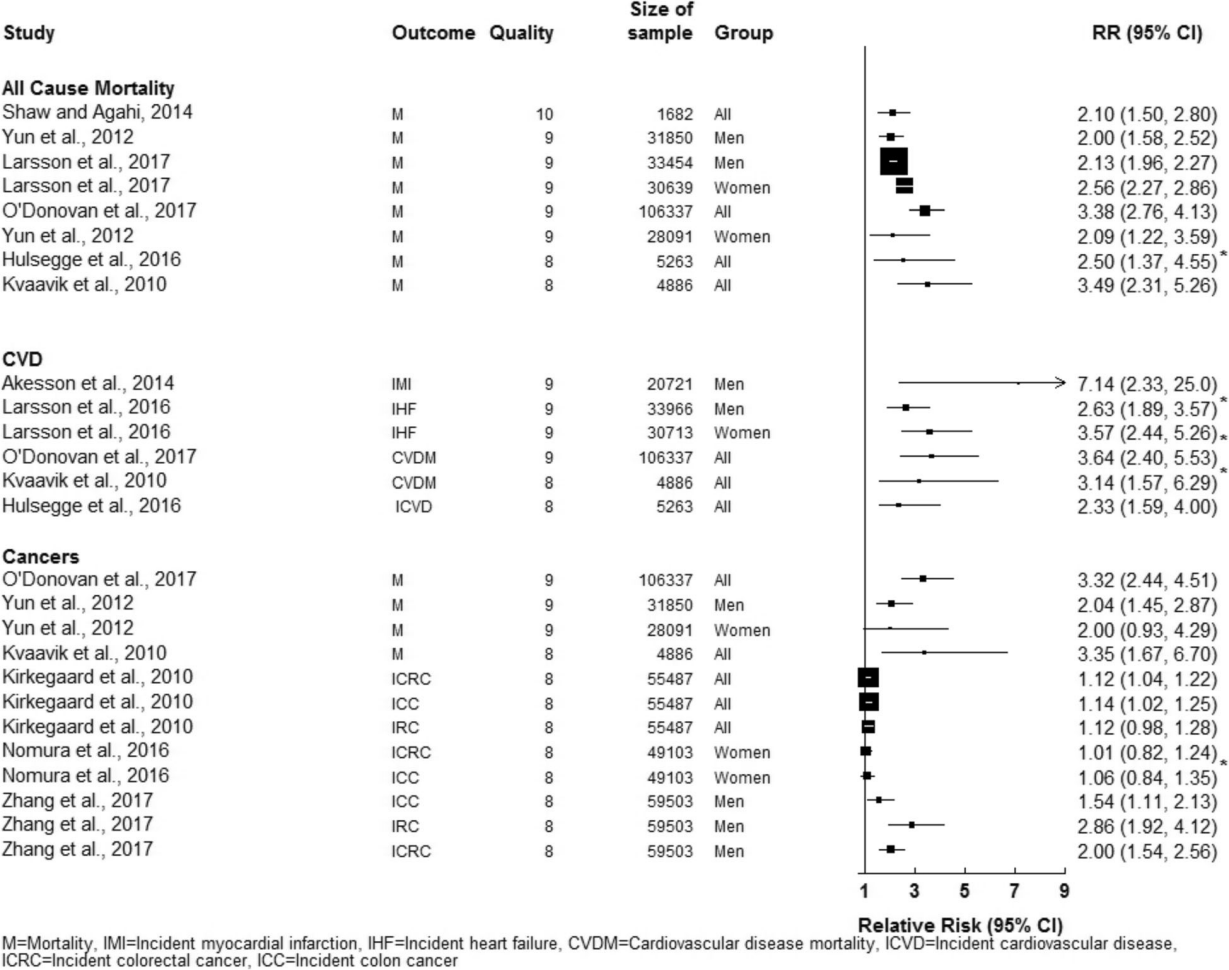
Forest plot of risk comparing those who were categorised as healthiest compared to those who were categorised as least healthy

Figure 2 examines the risk relationship between those in each cohort that were categorised as the healthiest (reference group) compared to those in the least healthy category. The results and evidence are quite strong that less healthy lifestyle behaviour puts one at a higher risk of death and cardiovascular disease incidence or mortality. Although the high-quality articles examining cancer outcomes displayed a similar trend to that seen in the all-cause mortality and CVD articles, the risk associations were not as large, and the findings for colorectal cancer were mixed.

## Discussion

Overall, we aimed to examine the association between how higher levels of physical activity in combination with additional positive lifestyle behaviours impacts cardiovascular disease, cancer, and all-cause mortality. Across the 25 eligible studies, those participants who reported higher levels of physical activity combined with achieving other health behaviour goals compared to those who were categorised as physically inactive and did not achieve other positive lifestyle goals, were at least half as likely to experience an incident CVD event, die from CVD, or die from any cause. These findings were consistent across participant age, sex, and study length of follow-up, and even after excluding lower quality studies. We observed a similar trend among the few studies which were restricted to cancer outcomes. Although limited, evidence from high quality cohorts suggests that the categorisation of multiple lifestyle risks groups, and thus the group someone is categorised into, is strongly predictive of long-term mortality and cardiovascular and cancer morbidity.

To date, there have been few high-quality studies examining combined effects of physical activity and lifestyle risk factors on cardiovascular diseases, cancers, and mortality risk. We found that most studies were limited by bias attributable to residual confounding, reverse causality by pre-existing disease, and measurement error with self-report data. In most prospective studies examined, physical activity assessed at baseline was used to investigate the association of activity with health outcomes many years later. As a result, these studies do not accurately characterise activity during long periods of follow-up, since measurement error could occur at the time of assessment, and physical activity behaviours may change over time [40, 41]. Moreover, while measurement error may be less of a concern for other behavioural risk factors (except diet), changes over time are still common, and these errors may result in regression dilution bias. Regression dilution bias in these studies could have resulted in the attenuation in the estimates of the risk associations between behavioural risk factors assessed at baseline and the health outcome of interest [41]. Resurveys on a representative sample of study participants can be used to correct for regression dilution [40], and allow behavioural risk factor information to more accurately reflect the “usual” exposure levels over the follow-up period [41].

Additionally, we found that across studies, reporting of physical activity has been heterogeneous. Physical activity was measured in different contexts and environments, and generally included different scores for intensity, frequency and duration measurements through self-report. Without standardised agreement across publications, readers face several challenges in determining the implications and impact of the associations between lifestyle risk factors and disease outcomes [42].

All studies included in this review assessed multiple behavioural risk factors using co-occurrence methodologies. These methods have limitations as they require dichotomisation of lifestyle behaviours into ‘risky/unhealthy’ versus ‘not risky/healthy’ [6], and thereby limit conclusions that can be made on a dose response. In addition, co-occurrence methodologies provide no indication of whether the co-occurrence of behaviours is, in part or in whole, the result of the association between the behaviours [6]. Future research needs to explore the use of new analysis approaches, such as cluster analysis, to examine the association of multiple behavioural risk factors on health outcomes. For example, cluster analysis is not restricted to dichotomous behaviour categorisation, and allows the identification of association patterns using multiple levels for each variable, thereby providing answers on dose response [1].

Our findings suggest that health researchers, policy makers, and clinicians need to shift their focus from the independent effects to the combined effects of lifestyle behaviours on disease outcomes. Further investment in high quality research addressing this objective is warranted. Traditionally, the UK Department of Health has not adopted a holistic approach to preventive medicine [1], perhaps because policies on lifestyle risk factors have been separated into individual risks. The resulting focus has been on independent risk factors and their impact on diseases, rather than how multiple lifestyle risk factors are jointly distributed across the population, and how these impact disease outcomes [1].

### Limitations

Despite our rigorous title and abstract screening protocol, search terms for physical activity may have been omitted from the title and abstract and thus gone undetected. This is possible because, if a study focused predominately on smoking and alcohol, although physical activity could have been included in the full-text, it may not have been mentioned at the abstract level because this exposure was not one of the main exposures of interest. Additionally, each observational study adjusted by using different covariates, measuring exposures differently, and had different follow-up times. This may have led to the high heterogeneity observed in our review, thereby limiting our ability to conduct a meta-analysis. Given the many differences in how each study categorised and measured lifestyle behaviours, as well as, the differences in the number of behaviours included in each study, we were unable to compute an I^2^ statistic which would have provided a useful measure of the variation across these studies rather than that of chance.

Across all studies, physical activity was measured by using self-report or structured interviews. Therefore, recall bias within the reviewed results must be considered. We performed detailed data extraction of all included studies, and completed the NOS to assess study quality. Future work should apply more sophisticated and objective physical activity measurement methods. Also, there was little consistency across studies regarding how the frequency, intensity and duration of physical activity were recorded. Limiting this review to observational research, which in many cases only reported physical activity at baseline, allowed for conclusions to only consider physical activity during the time of examination. The re-measurement or continuous monitoring of physical activity levels between exposure measurement and outcome occurrence would allow for more accurate conclusions, and should be a focus of future work.

## Conclusions

In summary, this was the first global systematic review of adults to examine the combined effect of physical activity with other positive lifestyle behaviours and its relationship with cardiovascular disease, cancer, and mortality from any cause. High levels of physical activity when combined with other positive lifestyle choices are associated with better health outcomes. Physical activity is only one part of multifaceted lifestyle behaviours, and applying new approaches to studying the complex relationships between multiple behavioural risk factors, including physical activity should be a priority.

## Additional files

Additional file 1: Physical activity + risk factor(s) and incidence and mortality of non-communicable diseases. Data extraction form. (PDF 72 kb)

Additional file 2: Search Strategy. (DOC 63 kb)

Additional file 3: EMBASE. Search terms. (PDF 59 kb)

## Data Availability

Not applicable. However, if the reader wishes to perform the search again they can use the search strategy found in the supplementary materials.

## Abbreviations

CHD: coronary heart disease
CI: confidence interval
CVD: cardiovascular disease
HR: hazard ratio
NOS: Newcastle-Ottawa Scale
PRISMA: Preferred Reporting Items for Systematic Reviews and Meta-Analyses
RR: relative risk
UK: United Kingdom
