# Supplementary material for: The impact of physical activity and an additional behavioural risk factor on cardiovascular disease, cancer and all-cause mortality: a systematic review
Source: BMC Public Health. 2019 Jul 8;19:900. doi: 10.1186/s12889-019-7030-8 (PMC6615183; doi:10.1186/s12889-019-7030-8)
Supplement: Supplementary file 1 — Physical activity + risk factor(s) and incidence and mortality of non-communicable diseases. Data extraction form. (PDF 72 kb) [file 12889_2019_7030_MOESM1_ESM.pdf]

APPENDIX 1

Physical activity + risk factor(s) and incidence and mortality of non-communicable diseases

Data extraction form

Name of reviewer:

Date of extraction:

Section 1

Name of paper:

Year of paper:

Section 2

Inclusion (Write yes if conforms to all criteria above)

Confirm eligibility:

Comments:

Section 3 study methods

Type of study:

Total study duration:

Method used to cluster/group lifestyle behaviours:

Section 4 participants

Number:

Setting (community/hospital/both):

Outcome:

Mean Age:

Gender breakdown:

Country:

Section 5 interventions

<u>Intervention 1</u>

Title of intervention:

Details of intervention:

Follow-up linked to medical records (y/n):

Years of follow-up:

Comments:

Section 6 outcomes

For each outcome of interest

Outcome Title 1: All-cause Mortality

Length of follow up:

Time point collected:

Time point reported:

Outcome definition:

Outcome Title 2: **NCD**

Length of follow up:

Time point collected:

Time point reported:

Outcome definition:

Section 7 results

<u>Outcome Title 1</u> ***<u>All-cause Mortality</u>***

Comments

Sample size for this outcome			
Number of missing participants at follow-up			
Events:			
Specify time units (eg days)			
Any other data			
Any comments on measurement of this outcome			

<u>Outcome Title 2:</u> ***<u>NCD's</u>***

Comments

Sample size for this outcome			
Number of missing participants at follow-up			
Events:			
Specify time units (eg days)			
Any other data			
Any comments on measurement of this outcome			

Section 8

Funding source:

Key conclusions of authors:

Other comments:

What do we need to ask the authors?:

Also need to add in

Statistical methods (adjustment for baseline values, dilution regression applied, handling of missing data?)

Quality of exposure measures (reported validity, face validity?)

Quality of outcome measures

Type of exposure measure (continuous, binary)
