# Supplementary material for: The impact of physical activity and an additional behavioural risk factor on cardiovascular disease, cancer and all-cause mortality: a systematic review
Source: BMC Public Health. 2019 Jul 8;19:900. doi: 10.1186/s12889-019-7030-8 (PMC6615183; doi:10.1186/s12889-019-7030-8)
Supplement: Supplementary file 3 — EMBASE. Search terms. (PDF 59 kb) [file 12889_2019_7030_MOESM3_ESM.pdf]

EMBASE

1. (health$ adj2 (diet$ or eating or food or foods)).ti,ab.
2. (unhealth$ adj2 (diet$ or eating or food or foods)).ti,ab.
3. fruit/ or vegetable/
4. (fruit$ adj2 (eat or eats or eating or intake or consum$ or increas$ or portion$ or serving$ or frequenc$ or number$ or preference$ or choice$)).ti,ab.
5. (vegetable$ adj2 (eat or eats or eating or intake or consum$ or increas$ or portion$ or serving$ or frequenc$ or number$ or preference$ or choice$)).ti,ab.
6. "5 a day".mp.
7. "five a day".ti,ab.
8. (junk food or fast food).ti,ab.
9. ((decreas$ or reduc$ or discourag$ or limit$ or lessen or eat$ less) adj2 (salt or sodium or fat or sugar$)).ti,ab.
10. ((salt or sodium or fat or sugar$ or calor* or energy) adj2 (intake or consum*)).ti,ab.
11. (food adj (choice$ or frequenc$ or select$)).ti,ab.
12. feeding behavior/
13. eating habit/ or food preference/
14. exp *diet therapy/
15. *obesity/
16. caloric intake/
17. 1 or 2 or 3 or 4 or 5 or 6 or 7 or 8 or 9 or 10 or 11 or 12 or 13 or 14 or 15 or 16
18. sedentary lifestyle/
19. (physical$ adj3 (fit$ or train$ or activ$ or inactiv$ or endur$)).ti,ab.
20. (exercis$ adj3 (fit$ or train$ or activ$ or endur$)).ti,ab.
21. ((promot$ or uptak$ or encourag$ or increas$ or start$ or adher$) adj3 (exercis$ or gym$ or sport$ or fitness)).ti,ab.
22. (sedentary behaviour$ or sedentary behavior$ or sedentary lifestyle$ or sedentariness).ti,ab.
23. ((decreas$ or reduc$ or discourag$) adj3 (sedentary or deskbound)).ti,ab.
24. *fitness/
25. ((watch$ or view$) adj2 (tv or television)).ti,ab.
26. (sport$ or walk$ or running or jogging or bicycling or biking or swimming).ti,ab.
27. (active adj (travel$ or transport$ or commut$)).ti,ab.
28. exp *exercise/
29. exp *physical activity/
30. 18 or 19 or 20 or 21 or 22 or 23 or 24 or 25 or 26 or 27 or 28 or 29
31. *smoking/
32. (smoking or antismoking or anti-smoking).ti,ab.
33. (smoker or smokers).ti,ab.
34. tobacco/ or tobacco.ti,ab.
35. *tobacco/
36. 31 or 32 or 33 or 34 or 35
37. drinking behavior/
38. alcoholic beverage/
39. (beer or wine$ or cider or alcopop$ or spirit or spirits).ti,ab.
40. alcohol$.ti,ab.

41. (drink$ adj2 (binge or excessive or harm$ or heavy or misus$ or abus$ or consum$)).ti,ab.

42. (intoxicat$ or inebriat$ or drunk$).ti,ab.
43. 37 or 38 or 39 or 40 or 41 or 42
44. (30 and 17) or (30 and 36) or (30 and 43)
45. (random$ or placebo$ or single blind$ or double blind$ or triple blind$).ti,ab.
46. 44 and 45
47. (exp animals/ or nonhuman/) not human/
48. (editorial or letter or note or conference*).pt.
49. 47 or 48
50. 46 not 49
51. limit 50 to yr="2009 -Current"
52. exp cohort analysis/ or exp longitudinal study/ or exp prospective study/ or exp follow up/

53. (exp animals/ or nonhuman/) not human/
54. (editorial or letter or note or conference*).pt.
55. 53 or 54
56. 52 not 55
57. 44 and 56
58. limit 57 to yr="2009 -Current"
59. 51 or 58
60. exp juvenile/ not (exp juvenile/ and exp adult/)
61. 59 not 60

MEDLINE

1. (health$ adj2 (diet$ or eating or food or foods)).ti,ab.
2. (unhealth$ adj2 (diet$ or eating or food or foods)).ti,ab.
3. fruit/ or vegetables/
4. (fruit$ adj2 (eat or eats or eating or intake or consum$ or increas$ or portion$ or serving$ or frequenc$ or number$ or preference$ or choice$)).ti,ab.
5. (vegetable$ adj2 (eat or eats or eating or intake or consum$ or increas$ or portion$ or serving$ or frequenc$ or number$ or preference$ or choice$)).ti,ab.
6. "5 a day".mp.
7. "five a day".ti,ab.
8. (junk food or fast food).ti,ab.
9. ((decreas$ or reduc$ or discourag$ or limit$ or lessen or eat$ less) adj2 (salt or sodium or fat or sugar$)).ti,ab.
10. ((salt or sodium or fat or sugar$ or calor* or energy) adj2 (intake or consum*)).ti,ab.
11. (food adj (choice$ or frequenc$ or select$)).ti,ab.
12. feeding behavior/
13. food habits/ or food preferences/
14. nutrition therapy/ or exp diet therapy/
15. obesity/ or overweight/
16. Energy Intake/
17. 1 or 2 or 3 or 4 or 5 or 6 or 7 or 8 or 9 or 10 or 11 or 12 or 13 or 14 or 15 or 16
18. (physical$ adj3 (fit$ or train$ or activ$ or inactiv$ or endur$)).ti,ab.
19. (exercis$ adj3 (fit$ or train$ or activ$ or endur$)).ti,ab.

20. ((promot$ or uptak$ or encourag$ or increas$ or start$ or adher$) adj3 (exercis$ or gym$ or sport$ or fitness)).ti,ab.
21. ((decreas$ or reduc$ or discourag$) adj3 (sedentary or deskbound)).ti,ab.
22. (sedentary behaviour$ or sedentary behavior$ or sedentary lifestyle$ or sedentariness).ti,ab.
23. sedentary lifestyle/
24. ((watch$ or view$) adj2 (tv or television)).ti,ab.
25. (sport$ or walk$ or running or jogging or bicycling or biking or swimming).ti,ab.
26. (active adj (travel$ or transport$ or commut$)).ti,ab.
27. physical fitness/
28. exp Recreation/ or leisure activities/
29. exp Exercise Therapy/ or Exercise/
30. running/ or jogging/ or swimming/ or walking/ or yoga/
31. 18 or 19 or 20 or 21 or 22 or 23 or 24 or 25 or 26 or 27 or 28 or 29 or 30
32. exp smoking/
33. (smoking or antismoking or anti-smoking).ti,ab.
34. (smoker or smokers).ti,ab.
35. tobacco/ or tobacco.ti,ab.
36. 32 or 33 or 34 or 35
37. exp Alcohol Drinking/
38. exp Alcoholic Intoxication/
39. exp Alcoholic Beverages/
40. exp Drinking Behavior/
41. (beer or wine$ or cider or alcopop$ or spirit or spirits).ti,ab.
42. alcohol$.ti,ab.
43. (drink$ adj2 (binge or excessive or harm$ or heavy or misus$ or abus$ or consum$)).ti,ab.
44. (intoxicat$ or inebriat$ or drunk$).ti,ab.
45. 37 or 38 or 39 or 40 or 41 or 42 or 43 or 44
46. (31 and 17) or (31 and 36) or (31 and 45)
47. randomized controlled trial.pt.
48. controlled clinical trial.pt.
49. randomized.ab.
50. randomly.ab.
51. placebo.ab.
52. clinical trials as topic.sh.
53. trial.ti.
54. 47 or 48 or 49 or 50 or 51 or 52 or 53
55. 46 and 54
56. exp animals/ not humans.sh.
57. (comment or editorial or letter).pt.
58. 56 or 57
59. 55 not 58
60. limit 59 to yr="2009 -Current"
61. (cohort adj (study or studies)).tw.
62. cohort analy$.tw.
63. ("follow up" adj (study or studies)).tw.
64. (observational adj (study or studies)).tw.

65. (descriptive adj (study or studies)).tw.
66. (epidemiologic$ adj (study or studies)).tw.
67. (longitudinal or retrospective or prospective).tw.
68. cohort studies/ or longitudinal studies/ or retrospective studies/ or prospective studies/ or follow-up studies/ or epidemiologic studies/
69. 61 or 62 or 63 or 64 or 65 or 66 or 67 or 68
70. exp animals/ not humans.sh.
71. (comment or editorial or letter).pt.
72. 70 or 71
73. 69 not 72
74. 46 and 73
75. limit 74 to yr="2009 -Current"
76. 60 or 75
77. exp child/ not (exp child/ and exp adult/)
78. 76 not 77
